# The Book World of Medicine and Science

**Published:** 1896-11-21

**Authors:** 


### THE BOOK WORLD OF MEDICINE AND SCIENCE.

Guide to the Examination in Practical Chemistry for the Conjoint Board. By Percy A. E. Richards, F.I.C., F.C.S. (London: Baillière, Tindall, and Cox. 1896.)

This is but a little book of 57 pages. But the subject is not a very large one, the knowledge of practical chemistry demanded by the Conjoint Board being neither very wide nor very deep, and the book before us seems quite sufficient to guide students through the difficulties of the "practical," supposing them to know the general subject. At the end of the book a few useful pages of equations are given, showing, in their proper formula, the reactions taking place in the various processes described. We can certainly commend this "guide" to students as well fitted for its purpose.

Observations on Congenital Dislocation of the Hip. By Bernard E. Brodhurst, F.R.C.S. Third Edition. (London: J. and A. Churchill. 1896. Price 2s. 6d.)

In the thirty-three pages of which this book consists Mr. Brodhurst gives a useful description of the causes and the treatment of a condition which is undoubtedly too often overlooked in the early months of infantile life. "Congenital" dislocation of the hip may arise from displacements occurring at birth through traction or through muscular action. With a breech presentation the thighs are flexed upon the abdomen in such a manner that the head of the femur presses on the posterior and inferior portion of the capsule of the joint, and while in this position the slightest force in traction, whether with the finger of the accoucheur or with the blunt hook in the bend of the thigh, will displace the femoral head. This will then lie on the brim of the acetabulum, but extension of the limb is sufficient to displace it beyond the brim and produce complete dislocation. It may also be caused by extension in a foot case. Dislocation may also be produced actually *in utero* by spasmodic action resulting from sudden shock or jar, and in this case it is apt to be accompanied by considerable arrest of development. Cases of dislocation arising from inflammation and destruction of the joint in the early months of life are sometimes improperly included among congenital dislocations; and, lastly, there are cases which are true malformations and are often associated with other serious deformities. As a matter of diagnosis it is well also to bear in mind that form of rickets in which the neck of the femur forms a right angle with the shaft, the trochanter being thus brought above Nélaton's line, for this is not infrequently mistaken for congenital dislocation. In the first class of cases, viz., those which occur at birth, the head may be replaced within two years, or even after three or four years have elapsed, without section of the muscles; but in the cases met with later on in life it is impossible to overcome retraction of the trochanteric muscles and the adductors of the thigh by extension alone; and Mr. Brodhurst advises free division of these muscles together with subcutaneous gouging out from the acetabulum of any fatty or other substances by which its cavity may have become filled.

Public Health in European Capitals. By Thomas Morison Legge, M.A., M.D. (Oxon.), D.P.H. (London: Swan Sonnenschein and Co., Paternoster Square, 1896. Price 3s. 6d.)

Preventive medicine may well claim a first place among modern social advances. Here in England we have looked to our drains and our water supplies, so that the general death-rate, along with the particular mortality begotten of bad drains and polluted water, has steadily fallen from decade to decade during the past half century. With all this improvement, however, he would be a rash man who would assert that hygiene is yet beyond the days of its weakling infancy, or that, despite our acknowledged superiority in many points, we have not yet a great deal to learn from the experience of other countries. Hence it is with pleasure we turn to a little volume lately issued by Dr. Legge under the title, "Public Health in European Capitals." Its twelve chapters treat of the sanitary administration, water supply, drainage, housing of the poor, notification of infectious diseases, public abattoirs, control of milk and meat supplies, and other items of sanitary interest in Berlin, Paris, Brussels, Christiania, Stockholm, and Copenhagen. Much of the mass of material thus gathered together has already appeared in print elsewhere. The chapters on the Scandinavian capitals, for instance, are reprints from the pages of the *Lancet*, whilst others are drawn from various medical journals. The chief merit of this little book, which does not profess to deal with minutiæ, lies in the fact of its being the sole outcome of personal inquiry and of what has been actually seen by the author. Dr. Legge lays stress on a fundamental difference between the mode of administering public health regulations in England and in the continental capitals. In general, it may be said that the independent position of the health service which is found in Great Britain does not exist to anything like the same extent in Germany, nor are the laws dealing with public health matters so complete or so well arranged. But notwithstanding our enjoyment of superior administrative advantages, as already hinted, the foreigner can teach us many useful lessons, both private and municipal. This is especially evident in the construction and the working of the Berlin hospitals, asylums, night shelters, people's kitchens, and public markets, as well as the thorough management of such matters as meat inspection, milk control, water filtration, the care of foundlings and orphans, sewage farms, and street cleansing. A good account is given of the success of the sewage farms around the German capital, where all sewage is utilised, instead of being cast into the sea as in London, or drained in the nearest river as at Paris. The description of the water supply of the latter capital is worth careful study as a monumental instance of municipal muddle and of consequent disaster to the health and the lives of consumers. Space does not permit us to dwell upon the many useful comparisons suggested by the further perusal of this book. While Dr. Legge's work is obviously too small for general reference, it is, nevertheless, both interesting and valuable as a means of study and information. Its literary style is clear and smooth, qualities that are of special value to the reader in grasping the large amount of material condensed between the covers of this little volume of 198 pages. It should be in the hands of everyone who is interested in preventive medicine, a subject that is now of universal and cosmopolitan importance.

